# Association of mean platelet volume/lymphocyte ratio with inflammation in non-dialysis patients with chronic kidney disease stages 1–4: A retrospective study

**DOI:** 10.3389/fimmu.2022.1041356

**Published:** 2022-11-18

**Authors:** Bei Xu, Yamei Zhang, Gang Chen, Jiafu Feng, Lingling Gan

**Affiliations:** Department of Clinical Laboratory, Mianyang Central Hospital, School of Medicine, University of Electronic Science and Technology of China, Mianyang, China

**Keywords:** chronic kidney disease, mean platelet volume/lymphocyte ratio, inflammation, kidney, non-dialysis

## Abstract

**Objective:**

The mean platelet volume/ratio (MPVLR) is recognized as a novel marker of inflammation. We evaluated whether the MPVLR is associated with inflammation in non-dialysis patients with chronic kidney disease (CKD) stages 1–4.

**Methods:**

A total of 402 non-dialysis patients with CKD stages 1–4 were included. The indicators of hematological, renal function (urea, serum creatinine [Scr], estimated glomerular filtration rate [eGFR], and urine albumin to creatinine ratio [ACR]) and the markers of inflammation (high-sensitivity C-reactive protein [hsCRP] and fibrinogen [FIB]) were recorded. The MPVLR values at different CKD stages were analyzed. Next, based on the absence (hsCRP level < 5 mg/L) and presence (hsCRP level > 5 mg/L) of inflammation, the patients were categorized, and the differences in indices between the two groups were observed. The patients were divided into two groups based on the median MPVLR value (6.39) at admission. The laboratory indices of patients with CKD were compared. Simultaneously, a correlation analysis was performed to identify the association between the MPVLR and each parameter. A binary logistic regression analysis was performed to test whether the MPVLR was associated independently with the presence of inflammation in non-dialysis patients with CKD. The receiver operating characteristic (ROC) curve was used to analyzed diagnostic performance of the MPVLR in evaluating the inflammation of non-dialysis patients with CKD stages 1-4.

**Results:**

The MPVLR was higher in patients with CKD stages 3–4 than in those with CKD stages 1 and 2. Significant differences in urea, Scr, eGFR, ACR, lymphocyte (LYM), red blood cell (RBC), hemoglobin (HGB), RBC distribution width (RDW-CV), MPVLR, and FIB values were observed between the groups with and without inflammation. The patients with a higher MPVLR had higher urea, Scr, ACR, WBC, neutrophils (NEU), RDW-CV, platelet distribution width (PDW), mean platelet volume (MPV), and hsCRP values and lower eGFR, LYM, RBC, HGB, and platelet (PLT) values. The MPVLR showed a positive correlation with age, urea, Scr, WBC, NEU, RDW-CV, PDW, MPV, and hsCRP values and a negative correlation with the eGFR, LYM, RBC, HGB, and PLT values. A logistic analysis revealed that the MPVLR was associated independently with the presence of inflammation in non-dialysis patients with CKD, after adjustment for the confounding factors (odds ratio = 1.020; *P* = 0.024). Furthermore, MPVLR exhibited a modest diagnostic performance for the assessment of inflammation in non-dialysis patients with CKD stages 1-4, with an area under the curve (AUC) of 0.706, and the sensitivity, specificity being 46.2% and 83.2%, respectively.

**Conclusions:**

The MPVLR was associated independently with the presence of inflammation in non-dialysis patients with CKD and may be useful for monitoring inflammation.

## Introduction

In the past few decades, chronic kidney disease (CKD) has become a major public health problem that has been estimated to affect approximately 800 million people worldwide, and it also remains the tenth leading cause of death (1). CKD is defined as an abnormal kidney structure or function that persists for > 3 months (2). With the progression of the disease, the glomerular filtration rate (GFR) continues to decrease, followed by the accumulation of metabolic toxins in the body and disorders of the immune system. In patients with CKD, systemic inflammation is characterized by a low-grade inflammatory status (3). The levels of inflammatory cytokines, e.g., tumor necrosis factor-α (TNF-α) and interleukin (IL), in the systemic circulation have been shown to increase slightly to regulate the inflammatory response and to mediate some of their downstream effects through the positive acute-phase proteins, e.g., the C-reactive protein (CRP), fibrinogen (FIB), and albumin (4). Unlike the inflammation caused by bacteria, fungi, or viruses, this inflammation is mild and persistent, and patients do not show any obvious clinical manifestations of infection. In turn, this persistent low-grade inflammation has been shown to further aggravate kidney damage, leading to the occurrence and development of various complications of CKD, such as cardiovascular disease, malnutrition, and renal bone disease (5). Currently, there are no uniform standards, that is based mainly on the elevation of traditional inflammatory biomarkers, e.g., hypersensitive CRP (hsCRP), TNF-α, and IL for diagnosing low-grade systemic inflammation (6). Due to the expense, technical constraints, and the application primarily in scientific research, the clinical application of inflammatory markers, such as TNF-α and IL, has been limited in clinical settings. There has been a reduction in major adverse cardiovascular events with the use of inflammation-targeted therapy in patients with CKD, as shown in recent data from the CANTOS trial (7). Therefore, it is necessary to develop a simple, convenient, and inexpensive index to determine a slightly inflammatory state.

The complete blood count (CBC) is an inexpensive and credible test used widely to evaluate disease status, such as anemia, infection, hematologic malignancies, and coagulation disorders. Recently, the mean platelet volume-to-lymphocyte ratio (MPVLR), the ratio of mean platelet volume (MPV) to lymphocytes (LYMs) in the CBC, has received significant attention (8). It is considered as a novel inflammatory indicator and prognostic or diagnostic marker. A previous study showed that increased MPVLR values may be valuable in the diagnosis of pediatric acute appendicitis and may also help distinguish between uncomplicated and perforated appendicitis in children (9). In patients with an ascending thoracic aortic aneurysm, the MPVLR can be evaluated as a useful parameter in the emergency clinical approach for evaluating inflammatory activity (10). Ornek et al. (11) has shown that the MPVLR might predict the development of the coronary collateral circulation in patients with stable coronary artery disease. As an independent predictor, the MPVLR is significantly higher in patients with contrast-induced nephropathy which is one of the most important complications after invasive cardiovascular procedures, than in those without (12). Kocak et al. reported that MPVLR can be considered as a powerful and independent predictor of diabetic nephropathy in diabetic patients (13). An independent association with worse cardiovascular events (CVEs), rehospitalization for heart failure (HF), in-hospital death, and composite outcomes was found for the elevated MPVLR on admission in patients with acute HF (14). Moreover, in patients with nonmetastatic clear cell renal cell carcinoma treated with nephrectomy, the MPVLR is a prognostic marker for overall survival since a high MPVLR was shown to be independently associated with a higher long-term overall mortality (15). Nonetheless, there have been no reports that focused on the association between the MPVLR and inflammation in patients with CKD. Therefore, in this retrospective cohort study, we investigated whether the MPVLR can be used as a reliable marker for assessing inflammation in CKD. We hypothesized that the MPVLR may to a certain degree, reflect the systematic inflammation of patients with CKD.

## Materials and methods

### Ethics statements

This study was approved by the Ethical Review of Medical Technology committee of the Mianyang Central Hospital (approval number: P2020040).

### Study design and participants

This retrospective cohort study recruited 402 patients with CKD stages 1–4 who were not on dialysis at the Mianyang Central Hospital, School of Medicine, University of Electronic Science and Technology of China, from January 2018 to December 2021. The inclusion criterion for patients with CKD was those who met the diagnostic criteria of CKD as outlined in the Kidney Disease Improving Global Outcomes Clinical Practice Guidelines for the Evaluation and Management of Chronic Kidney Disease, 2012 (2). The exclusion criteria were: 1) age < 18 years; 2) the presence of hematologic diseases, carcinoma, metabolic or cardiovascular disorders, endocrinopathy, autoimmune abnormalities, and mental illness; 3) a recent history of blood transfusion, infection, or trauma; 4) pregnancy; 5) being administered vitamins, and anti-inflammatory, anti-depressant, and immune suppressor drugs.

According to the 2012 CKD Guidelines, the patients were divided into several subgroups based on the estimated GFR (eGFR): CKD stage 1, ≥ 90 mL/(min·1.73 m^2^); CKD stage 2, 60–89 mL/(min·1.73 m^2^); CKD stage 3, 30–59 mL/(min·1.73 m^2^); and CKD stage 4, 15–29 mL/(min·1.73 m^2^).

### Data collection and laboratory methods

All the baseline characteristics and indices were obtained from the laboratory and hospital information systems. The results of the first CBC test were recorded. Venous blood samples were obtained from each patient in the early morning after fasting for 8–12 hours on admission. The white blood cell (WBC) count, absolute number of LYMs, neutrophils (NEU) count, red blood cell (RBC) count, hemoglobin (HGB) level, red blood cell distribution width (RDW-CV), platelet (PLT) count, platelet distribution width (PDW), and MPV were analyzed using the SYSMEX XN-9000 automatic analyzer (Sysmex Corporation, Kobe, Japan). The MPVLR was calculated using the following formula: MPVLR = MPV/LYM. The serum levels of urea, serum creatinine (Scr), and hsCRP were detected using the Labospect™ 008 automatic analyzer (Hitachi, Japan). The eGFR was calculated using the following formulas: eGFR = 175 × Scr^-1.234^ × age^-0.179^ (for men) and eGFR = 175 × Scr^-1.234^ × age^-0.179^ × 0.79 (for women). The plasma FIB levels were detected using the CS-5100 automatic coagulation analyzer (Sysmex Corporation). Urine samples (10 mL) were collected for the assessment of proteinuria. The urine albumin-to-creatinine ratio (ACR) was determined using the Biosystems A25 (BioSystems, Lexington, MA, USA). Internal quality control procedures and an external quality assessment scheme were used to ensure the quality of the test results. All the analyses were conducted within two hours of blood sample collection.

The reference interval for hsCRP that was obtained using this laboratory detecting system was 0–5 mg/L. For the purposes of this study, patients with an hsCRP level < 5 mg/L were categorized as the absence of inflammation group, whereas those with an hsCRP level > 5 mg/L were categorized as the presence of inflammation group. Patients with hsCRP levels of 5 mg/L were excluded to avoid introduction of bias into the study results.

Patients with CKD were also divided into the low MPVLR (MPVLR < 6.39) and high MPVLR (MPVLR ≥ 6.39) groups according to their median MPVLR. The hematological and biochemical indicators of the patients in the two groups were compared.

Our laboratory is certified according to the ISO 15189:2012 standard, and internal quality control procedures and external quality assessment schemes are performed regularly to validate the quality of the test results.

### Statistical analysis

The normal distribution of the continuous data was analyzed using the Kolmogorov–Smirnov test. The descriptive statistics were presented as proportions, means ± standard deviations, or median and interquartile ranges. The Chi-square test was used to compare the categorical variables between the groups and the student *t*-test was used to perform the pair-wise comparisons. The Mann–Whitney *U* test was used to assess the differences between the medians of the non-normally distributed data of the two groups. A one-way analysis of variance or the Kruskal–Wallis non-parametric test was used to perform the multiple group comparisons. The correlation of the MPVLR with the laboratory indices was obtained using the Spearman test. A binary logistic regression analysis was performed to identify the association between the MPVLR and inflammation in patients with CKD using the enter method. The dichotomized hsCRP variable was used as the dependent variable, the laboratory indices as the independent variable, and the hsCRP < 5 mg/L group as the reference. The potential confounders included age, urea, Scr, eGFR, LYM, RBC, HGB, and RDW-CV levels. The diagnostic performance was evaluated using a receiver operation characteristics (ROC) curve analysis and the optimal cut-off value was determined using the Youden method: Youden index (YI) = sensitivity + specificity − 1. The statistical analysis was performed using SPSS software (version 23.0; IBM Corp., Armonk, NY, USA). Statistical significance was set at *P* < 0.05 (two-sided tests).

## Results

### Patients’ demographic characteristics and clinical data

**Figure 1:**
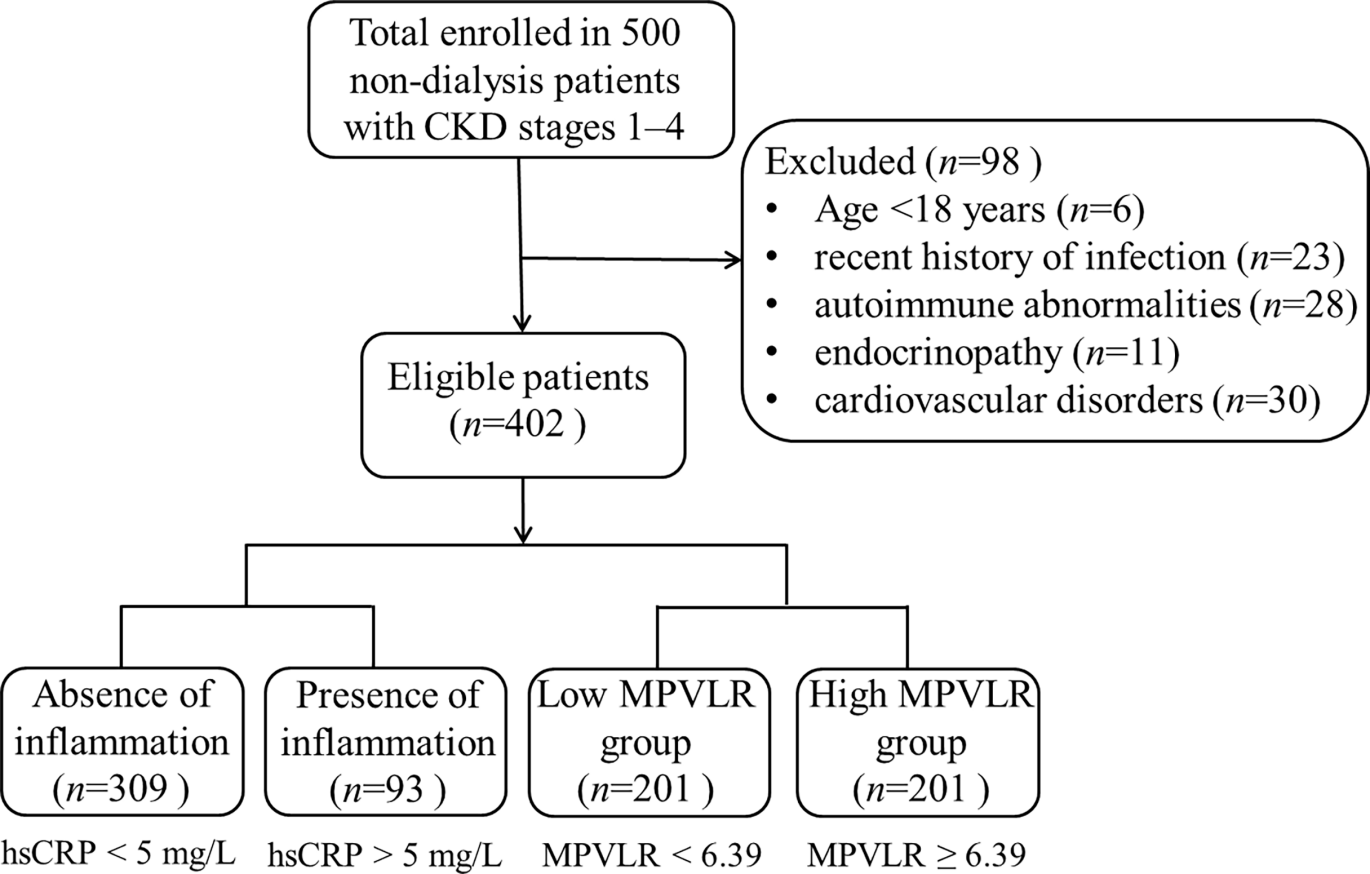
Flowchart of the study design and the patient categorization.

The laboratory data of the study population are presented in **Table 1**. The study included 402 patients with a median age of 47 years (range: 29–62 years) with 54.5% being male. Regarding the etiology of CKD, chronic glomerulonephritis, diabetic nephropathy, hypertensive nephropathy, and other undetermined diseases were present in 17.26%, 29.37%, 25.57%, 9.20%, and 18.60% of the patients, respectively. The patients with CKD were divided into three CKD stages: 73 patients with stage 1, 181 with stage 2, and 148 with stages 3–4. **Figure 1** shows the flowchart of the study design and the patients categorization.

**Table 1:** Patients’ demographic characteristics and clinical data.

Variable	CKD (*n* = 402)
Age,years	49.05 ± 16.83
Gender, n (%)(male)	219 (54.5)
Etiology of CKD, %	
Chronic glomerulonephritis	17.26%
Diabetic nephropathy	29.37%
Hypertensive nephropathy	25.57%
Other	9.20%
Undetermined	18.60%
CKD stage	
stage 1	18.16% (73 patients)
stage 2	45.02% (181 patients)
stage 3-4	36.82% (148 patients)
Blood biochemistry	
Urea (mmol/L)	5.98 (4.47-8.51)
Scr (μmol/L)	68.60 (53.20-88.83)
ACR (mg/g)	1054.04 (93.05-3333.58)
eGFR(mL/min/1.73 m^2^)	67.37 ± 23.32
WBC (×10^9^/L)	8.61 (6.54-11.42)
LYM (×10^9^/L)	1.83 (1.33-2.58)
NEU (×10^9^/L)	5.84 (3.93-8.38)
RBC (×10^12^/L)	4.28 ± 0.83
HGB (g/L)	128.25 ± 25.14
RDW-CV (%)	13.70 (12.90-15.10)
PLT (×10^9^/L)	201.50 (151.75-267.25)
PDW (%)	15.76 ± 4.09
MPV (fl)	11.68 ± 1.63
Inflammatory markers	
MPVLR	6.39 (4.34-9.18)
hsCRP(mg/L)	1.12 (0.32-4.44)
FIB (g/L)	4.33 ± 1.54

CKD, Chronic Kidney Disease; Scr, serum creatinine; ACR, urine albumin to creatinine ratio; eGFR, estimated glomerular filtration rate; WBC, white blood cell; LYM, lymphocytes; NEU, neutrophils; RBC, red blood cell; HGB, hemoglobin; RDW-CV, red blood cell distribution width; PLT, platelet; PDW, platelet distribution width; MPV, mean platelet volume; hsCRP, high-sensitivity C-reactive protein; FIB, fibrinogen; MPVLR=MPV/LYM.

**Figure 2:**
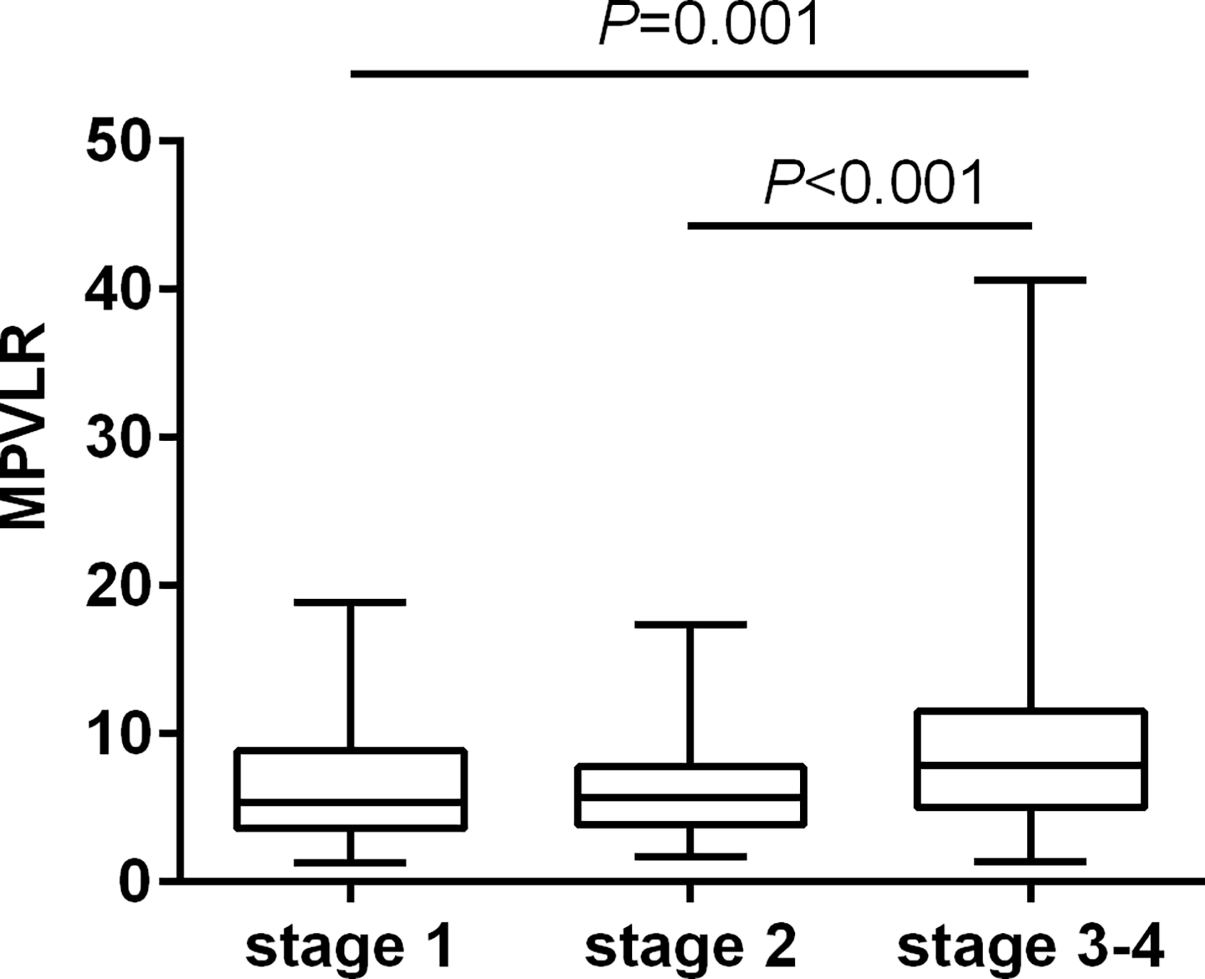
The level of MPVLR across CKD stages 1−4.

The results of multiple comparisons showed differences in the MPVLR across all the CKD stages (**Figure 2**). The patients in the subgroups of CKD stages 3 and 4 had higher MPVLR values than those in the other two subgroups (*P* < 0.001).

### Comparisons of clinical variables in the presence or absence of inflammation

The clinical parameters of the hsCRP groups are shown in **Table 2**. The analyses of the hematological and biochemical indices showed significantly increased urea, Scr, ACR, RDW-CV, MPVLR, and FIB values and decreased eGFR, LYM, RBC, HGB values in patients with inflammation, compared to those without inflammation. However, the WBC, PLT, PDW, and MPV values did not differ significantly between the groups.

**Table 2:** Comparisons of clinical variables in the presence or absence of inflammation.

Characteristics	absence of inflammation	presence of inflammation	*χ* ^2^/*Z*/*F*	*P*
	*n* = 309	*n* = 93		
Age,years	47.08 ± 16.08	55.59 ± 17.68	1.613	<0.001
Gender, n (%)(male)	169 (54.7)	50 (53.8)	0.025	0.875
Blood biochemistry				
Urea (mmol/L)	5.75 (4.39-7.83)	7.18 (5.08-10.75)	-3.711	<0.001
Scr (μmol/L)	65.60 (51.45-82.30)	79.80 (58.70-131.05)	-3.904	<0.001
eGFR (mL/min/1.73 m^2^)	70.55 ± 20.03	56.81 ± 24.48	2.245	<0.001
ACR (mg/g)	1012.45 (39.86-3075.69)	1682.68 (225.81-3742.76)	-2.190	0.029
WBC (×10^9^/L)	8.45 (6.52-11.45)	9.06 (6.57-11.32)	-0.435	0.663
LYM (×10^9^/L)	2.01 (1.37-2.71)	1.55 (1.03-2.25)	-3.678	<0.001
NEU (×10^9^/L)	5.64 (3.88-8.21)	6.20 (4.12-8.71)	-1.825	0.068
RBC (×10^12^/L)	4.35 ± 0.74	4.02 ± 1.03	14.012	0.006
HGB (g/L)	130.45 ± 22.07	120.92 ± 32.48	23.814	0.009
RDW-CV (%)	13.60 (12.90-14.80)	14.00 (13.20-16.30)	-2.863	0.004
PLT (×10^9^/L)	202.00 (153.00-265.50)	197.00 (136.00-281.00)	-0.643	0.520
PDW (%)	15.83 ± 4.20	15.52 ± 3.71	4.535	0.514
MPV (fl)	11.68 ± 1.69	11.68 ± 1.43	4.883	0.995
Inflammatory markers				
MPVLR	5.89 (4.00-8.86)	7.55 (5.22-11.16)	-3.421	0.001
FIB (g/L)	4.14 ± 1.43	4.96 ± 1.72	5.141	<0.001

Scr, serum creatinine; eGFR, estimated glomerular filtration rate; ACR, urine albumin to creatinine ratio; WBC, white blood cell; LYM, lymphocytes; NEU, neutrophils; RBC, red blood cell; HGB, hemoglobin; RDW-CV, red blood cell distribution width; PLT, platelet; PDW, platelet distribution width; MPV, mean platelet volume; FIB, fibrinogen; MPVLR=MPV/LYM.

### Comparison of laboratory indices between the high and low MPVLR groups

As shown in **Table 3**, the patients in the high MPVLR group were more likely to be older than those in the low MPVLR group. They also had higher urea, Scr, ACR, WBC, NEU, RDW-CV, PDW, and MPV values and lower eGFR, LYM, RBC, HGB, and PLT values than those in the low MPVLR group. With regard the traditional inflammatory markers, the difference in the hsCRP levels between the two groups was statistically significant (*P* < 0.001), whereas there was no statistically significant difference in the FIB between the two groups (*P* = 0.509).

**Table 3:** Comparison of laboratory indexes between the high and low MPVLR groups.

Variable	MPVLR		*χ* ^2^/*Z*/*F*	*P*
	Low (<6.39)	High (≥6.39)		
	n = 201	n =201		
Age, years	44.31 ± 16.32	53.79 ± 16.02	0.445	<0.001
Gender, n (%)(male)	115 (57.2)	104 (51.7)	1.214	0.271
Blood biochemistry				
Urea (mmol/L)	5.62 (4.27-7.36)	6.96 (4.62-9.73)	-4.091	<0.001
Scr(μmol/L)	62.80 (50.35-79.05)	74.2 (55.35-100.75)	-4.374	<0.001
eGFR (mL/min/1.73 m^2^)	71.49 ± 20.21	63.26 ± 25.47	11.100	<0.001
ACR (mg/g)	1007.00 (23.83-3403.75)	1203.32 (157.86-3328.91)	-0.976	0.329
WBC (×10^9^/L)	7.12 (5.72-9.75)	9.89 (7.98-12.79)	-8.139	<0.001
LYM (×10^9^/L)	2.57 (2.24-3.26)	1.33 (1.00-1.56)	-16.709	<0.001
NEU (×10^9^/L)	5.06 (3.73-7.81)	6.23 (4.35-8.45)	-3.492	<0.001
RBC (×10^12^/L)	4.49 ± 0.74	4.07 ± 0.86	2.023	<0.001
HGB (g/L)	134.13 ± 22.52	122.36 ± 26.29	3.764	<0.001
RDW-CV (%)	13.60 (12.80-14.75)	13.90 (13.20-15.25)	-2.648	0.008
PLT (×10^9^/L)	246.00 (185.50-297.50)	164.00 (133.50-224.50)	-8.275	<0.001
PDW (%)	14.64 ± 3.87	16.88 ± 4.01	0.624	<0.001
MPV (fl)	11.02 ± 1.51	12.34 ± 1.48	0.172	<0.001
Inflammatory markers				
hsCRP(mg/L)	0.74 (0.24-2.61)	1.76 (0.48-7.02)	-4.521	<0.001
FIB (g/L)	4.38 ± 1.50	4.28 ± 1.59	0.048	0.509

MPVLR=MPV/LYM; Scr, serum creatinine; eGFR, estimated glomerular filtration rate; ACR, urine albumin to creatinine ratio; WBC, white blood cell; LYM, lymphocytes; NEU, neutrophils; RBC, red blood cell; HGB, hemoglobin; RDW-CV, red blood cell distribution width; PLT, platelet; PDW, platelet distribution width; MPV, mean platelet volume; hsCRP, high-sensitivity C-reactive protein; FIB, fibrinogen.

### Association of the MPVLR with variables

The spearman correlation analysis revealed that the MPVLR showed a positive correlation with age, urea, Scr, WBC, NEU, RDW-CV, PDW, MPV, and hsCRP and a negative correlation with eGFR, LYM, RBC, HGB, and PLT. However, the correlation between the MPVLR and gender, ACR, or FIB was not statistically significant (**Table 4**).

**Table 4:** Association of the MPVLR with variables.

Variable	MPVLR	
	*r*	*P*
Age	0.312	<0.001
Gender	0.058	0.246
Urea	0.268	<0.001
Scr	0.300	<0.001
eGFR	-0.247	<0.001
ACR	0.061	0.223
WBC	0.464	<0.001
LYM	-0.967	<0.001
NEU	0.175	<0.001
RBC	-0.298	<0.001
HGB	-0.261	<0.001
RDW-CV	0.181	<0.001
PLT	-0.511	<0.001
PDW	0.371	<0.001
MPV	0.463	<0.001
hsCRP	0.268	<0.001
FIB	-0.073	0.143

MPVLR=MPV/LYM; Scr, serum creatinine; eGFR, estimated glomerular filtration rate; ACR, urine albumin to creatinine ratio; WBC, white blood cell; LYM, lymphocytes; NEU, neutrophils; RBC, red blood cell; HGB, hemoglobin; RDW-CV, red blood cell distribution width; PLT, platelet; PDW, platelet distribution width; MPV, mean platelet volume; hsCRP, high-sensitivity C-reactive protein; FIB, fibrinogen.

### Association of indices and inflammation in patients with CKD

As shown in **Table 5**, in the unadjusted model analysis, age, urea, Scr, eGFR, LYM, RBC, HGB, RDW-CV, and MPVLR were associated significantly with the presence of inflammation. After adjusting for all the confounders, the MPVLR (adjusted odds ratio = 1.020, 95% confidence interval: 1.003–1.037, *P* = 0.024) was associated independently and significantly, with inflammation in patients with CKD.

**Table 5:** Association of indexes and inflammation in patients with CKD.

Variable	Univariate analysis		Multivariate analysis	
	Odds ratio (95% CI)	*P*	Odds ratio (95% CI)	*P*
Age	1.032 (1.017, 1.048)	<0.001	0.985 (0.969, 1.001)	0.061
Urea	1.077 (1.036, 1.119)	<0.001	0.981 (0.912, 1.054)	0.595
Scr	1.005 (1.002, 1.008)	<0.001	1.002 (0.998, 1.006)	0.378
eGFR	0.974 (0.963, 0.984)	<0.001	1.156 (0.995, 1.343)	0.058
WBC	1.011 (0.953, 1.073)	0.718		
LYM	0.619 (0.475, 0.806)	<0.001	0.723 (0.503, 1.040)	0.080
NEU	1.057 (0.990, 1.128)	0.098		
RBC	0.621 (0.466, 0.828)	0.001	0.972 (0.423, 2.236)	0.947
HGB	0.985 (0.976, 0.994)	0.002	1.003 (0.975, 1.031)	0.853
RDW-CV	1.228 (1.089, 1.386)	0.001	0.984 (0.935, 1.036)	0.546
PLT	0.999 (0.997, 1.002)	0.544		
PDW	0.982 (0.928 1.040)	0.540		
MPV	1.000(0.867, 1.152)	0.996		
MPVLR	1.049 (1.012, 1.087)	0.009	1.020 (1.003, 1.037)	0.024

Multivariate analysis adjusted for Age, Urea, Scr, eGFR, LYM, RBC, HGB, RDW-CV.

CKD, Chronic Kidney Disease; Scr, serum creatinine; eGFR, estimated glomerular filtration rate; ACR, urine albumin to creatinine ratio; WBC, white blood cell; LYM, lymphocytes; NEU, neutrophils; RBC, red blood cell; HGB, hemoglobin; RDW-CV, red blood cell distribution width; PLT, platelet; PDW, platelet distribution width; MPV, mean platelet volume; hsCRP, high-sensitivity C-reactive protein; FIB, fibrinogen; MPVLR=MPV/LYM.

### Diagnostic efficiency of MPVLR for inflammation assessment of CKD

**Figure 3:**
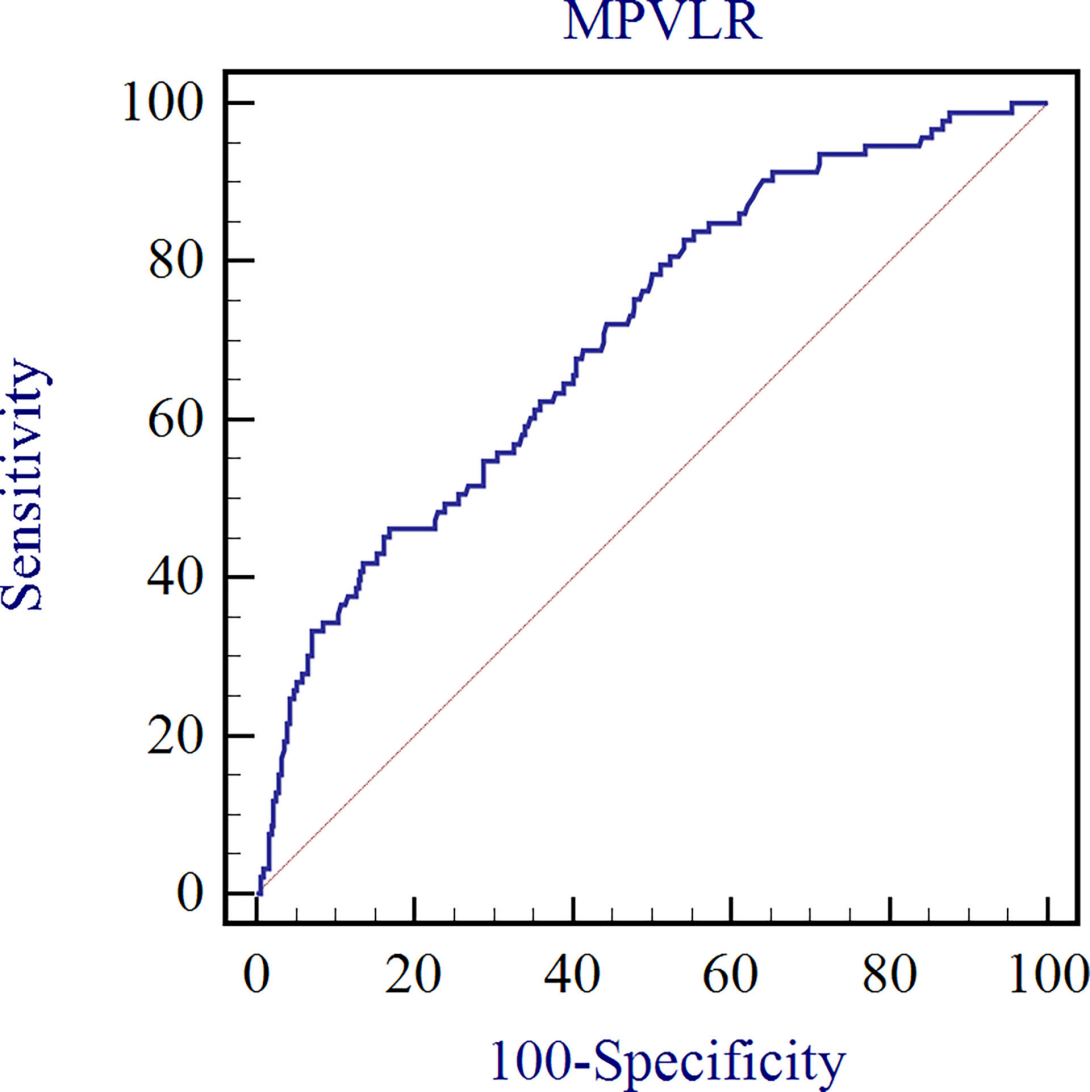
Diagnostic efficacy of MPVLR in the evaluation of the inflammation in non-dialysis patients with CKD stages 1–4.

The ROC curve analysis was performed to assess the diagnostic efficacy of MPVLR in the evaluation of the inflammation in non-dialysis patients with CKD stages 1–4 (**Figure 3**). The AUC was 0.706 and the MPVLR had a cut-off value of 9.64 with a sensitivity of 46.2% and a specificity of 83.2% (95% CI, 0.659–0.750).

## Discussion

Our results showed that the MPVLR value increased with a worsening of the renal function in non-dialysis patients with CKD stages 1–4. In contrast, patients with CKD stages 1–4, who had a higher hsCRP level, exhibited an elevated MPVLR value compared to those with a low hsCRP level. The logistic regression analysis revealed that the MPVLR was associated independently and significantly with inflammation after adjusting for the confounders, confirming our hypothesis. In addition, the MPVLR exhibited a modest diagnostic efficacy in the assessment of inflammation in non-dialysis patients with CKD stages 1–4. To our best knowledge, this is the first study to investigate the relationship between MPVLR and inflammation in non-dialysis patients with CKD stages 1–4.

Inflammation is a risk factor for accelerated CKD progression (16). Most patients with CKD stage 5 require dialysis treatment, which can promote the acute upregulation of proinflammatory cytokines directly and create additional inflammatory stimulation mediated by infectious and thrombotic events indirectly (17). Therefore, our study excluded patients with CKD stage 5. Alleviating the inflammatory state is one of the most effective means of reducing complications and death. The use of the platelet-to-lymphocyte ratio (PLR) for evaluating inflammation in patients with end-stage renal disease has been documented previously (18–20). Nevertheless, PLT size has long been regarded as a more accurate indicator of the functional and activation status than the PLT count (21). The MPV is an important index of the PLT volume and reflects the PLT function and activity. There is strong evidence that larger PLTs express higher levels of prothrombotic substances and possess greater metabolic and enzymatic activity than smaller PLTs (22). Studies have reported that the MPV is elevated in various diseases, such as atrial fibrillation, cerebrovascular disease, and Behçet disease (23, 24), and can play important roles in the inflammatory function of PLTs (25). It may also be plausible to substitute the MPV with the PLT count in the PLR to form the MPVLR (21). The value of the MPVLR has been reported in various diseases. The MPVLR was significantly higher in patients with ascending thoracic aortic aneurysm than in those without. It can be evaluated as a useful parameter in the emergency clinical approach in the assessment of inflammatory activity (10). Inflammation is associated with an increased risk of thrombotic events, and in patients with acute deep vein thrombosis, an increased MPVLR has been shown to have a potential diagnostic value (26). Bozlu et al. reported that the MPVLR was significantly higher in children who underwent appendectomy than in those who did not; thus, it can help distinguish between uncomplicated and perforated appendicitis in children (9). However, little research has been conducted on the usefulness of the MPVLR in patients with CKD.

The hsCRP is a representative biomarker of systemic inflammation (27). The hsCRP level is < 5 mg/L in healthy people, and it increases with the deterioration of renal function in patients with CKD. Studies have reported that higher levels of inflammatory markers are associated with a faster CKD progression (19). Here, MPVLR values increased with CKD staging. Crucially, by grouping CKD patients with a cutoff point of 5 mg/L, those exhibiting inflammation with hsCRP levels above the cut-off value had higher MPVLR values. They also had higher urea, Scr, and ACR values and lower eGFR, RBCs and HGB values, indicating a worse renal function and nutritional status. Similarly, we also found that patients with a higher MPVLR had a worse renal function and stronger inflammation than those with a lower MPVLR, as exhibited by higher WBC, NEU, and hsCRP values. After adjusting for the confounding factors, the MPVLR remained a significant predictor of inflammation in patients with CKD. In the past decade, MPV has been investigated in various diseases. Moreover, decreased MPV values are present in high-grade inflammatory diseases, e.g., active systemic lupus erythematosus (28), the active phase of rheumatoid arthritis (29), and ulcerative colitis (30). In contrast, increased MPV values are correlated mainly with low-grade inflammation states, such as the irritable bowel syndrome (31), cellulitis (32), and obstructive sleep apnea syndrome (33). Furthermore, there are conflicting reports with respect to the changes in the MPV in patients with CKD. In a retrospective study involving 553 CKD patients in Korea, Ju et al. (34) found that the MPV was increased significantly with the progression of CKD (9.81 ± 0.13 *vs.* 10.34 ± 0.08 *vs.* 10.86 ± 0.09 *vs.* 11.19 ± 0.11, corresponding to CKD stages 2, 3, 4, and 5, respectively). In contrast, in a study on the routine blood parameters of 627 patients with CKD stages 3−5, Erken et al. (35) reported that a low MPV may be associated with worse renal function (9.88 ± 1.74, 9.88 ± 1.39, 9.34 ± 1.53, corresponding to CKD stages 3, 4, and 5, respectively). In the present study, those in the high MPVLR group with worse renal function had higher MPV levels, supporting the findings of Ju et al. These inconsistent findings may have been due to: 1) Ju’ study including CKD stages 2–5 while Erken’s study enrolled patients with CKD stages 3–5; 2) The latter study also included patients with CKD stage 5 who were on hemodialysis and chronic peritoneal dialysis. Regarding the LYM count, it has been suggested that a decreased LYM count may be correlated with increased inflammatory responses (36). In this study, we observed decreased LYM counts in patients with CKD in the high hsCRP and MPVLR groups, which provided further evidence that patients with higher MPVLR level experience stronger inflammation.

As one of the most accessible and fundamental types of examination, a routine blood test has long been recommended as an essential tool for the diagnosis of disease. The indicators that reflect the status of inflammation have been identified as procalcitonin (PCT) (37), high sensitive C-reactive protein (hsCRP) (27), and Serum Amyloid A (SAA) (38); moreover, in a clinic, the MPVLR can be obtained easily from blood analysis, with the advantages of being convenient, technically simpler, less time-consuming, economical, and requiring less blood. However, the present study was preliminary. In the future, the prediction model of the MPVLR on inflammation in patients with CKD needs to be further established to confirm the cut-off value, which can help clinicians assess the occurrence of inflammation in CKD patients by dynamically monitoring the changes of this index.

This study has some limitations. First, given the retrospective nature of the study, we were unable to obtain detailed treatment data and medication history which may have influenced the results of the study. Second, the MPVLR was recorded only at the first CBC test on admission, and whether the MPVLR changed over time, is unknown. Third, the patients enrolled in this study were from local urban areas, so they were not representative of the national population, which may limit the generalizability of these findings to other populations. Finally, as discussed in the literature, because the MPVLR may reflect an altered platelet-mediated immunity and a decreased LYM-mediated immune response, the connection between the MPVLR and inflammation in CKD remains unclear. It is recommended that prospective multicenter studies be conducted to further validate the value of the MPVLR for inflammation in patients with CKD.

## Conclusions

In non-dialysis patients with CKD, elevated MPVLR values were observed, which increased with a worsening renal function. Increased MPVLR values were observed in patients with inflammation compared to those without inflammation. Moreover, the MPVLR was associated independently and significantly with inflammation. Due to the advantages of being simple, inexpensive, and rapid, the MPVLR may be useful in clinical inflammation assessments in non-dialysis patients with CKD stages 1–4.

## Data availability statement

The raw data supporting the conclusions of this article will be made available by the authors, without undue reservation.

## Ethics statement

The studies involving human participants were reviewed and approved by Medical Ethics Committee of Mianyang Central Hospital. Written informed consent for participation was not required for this study in accordance with the national legislation and the institutional requirements.

## Author contributions

All authors: study conception and design. LG and BX: data collection, interpretation, and manuscript writing and revision. LG, YZ, and GC: data analysis. JF and BX: data curation and supervision. YZ, GC, and BX: project administration and funding acquisition. All authors contributed to the article and approved the submitted version.

## Funding

This research was financially supported by the Youth Innovation Research Project of Sichuan Medical Association (grant number Q21012), and Hospital-Level Project of Mianyang Central Hospital [2019YJ14].

## Acknowledgments

We would like to acknowledge Dr. Mengjun Zhou for assistance with statistics.

## Conflict of interest

The authors declare that the research was conducted in the absence of any commercial or financial relationships that may be construed as a potential conflict of interest.

## Publisher’s note

All claims expressed in this article are solely those of the authors and do not necessarily represent those of their affiliated organizations, or those of the publisher, the editors and the reviewers. Any product that may be evaluated in this article, or claim that may be made by its manufacturer, is not guaranteed or endorsed by the publisher.
